# Epidemioclinical profile of intimate partner abusers

**DOI:** 10.1192/j.eurpsy.2022.2216

**Published:** 2022-09-01

**Authors:** M. Kacem, R. Ben Soussia, N. Faouel, M. Boughattass, W. Bouali, A. Haj Mohamed, L. Zarrouk

**Affiliations:** 1 University Hospital of Mahdia, Tunisia., Psychiatry, mahdia, Tunisia; 2 hospital Tahar sfar Mahdia, Department Of Psychiatry Mahdia, Mahdia, Tunisia; 3 hospital Tahar sfar Mahdia, Department Of Psychiatry Mahdia, monastir, Tunisia

**Keywords:** profile, violence, women, abuser

## Abstract

**Introduction:**

Domestic violence is a critical global and social phenomenon.

**Objectives:**

- To describe the socio-demographic and clinical characteristics related to the abuser in the context of domestic violence. - To study risk factors for acting out in the abuser.

**Methods:**

We conducted a descriptive cross-sectional study related to male abusers of their wives who consulted the Forensic Medicine Department of Taher Sfar Hospital in Mahdia between January 2020 and October 2020 for a forensic examination.

**Results:**

We collected 67 cases of domestic violence out of a total of 688 female consultants. The age of the abuser exceeded 35 years in 84% of cases. The average age of the abusers was 33.8 years. Almost half of the abusers had a primary school education. In 43% of the cases, the abuser was unemployed or had a job with a salary below the minimum wage. We found an association between domestic violence and the unfavorable professional status of the spouse. Only in 6% of the cases did the abuser have a psychiatric disorder. He had a history of chronic alcoholism in 35% of the cases and the use of illicit substances (cannabis) in 9% of the cases. Approximately one out of every two abusers (48%) was under the influence of alcohol at the time of the violent act. Alcoholism was associated with all forms of domestic violence. He had a criminal history in 30% of cases.

**Conclusions:**

Our results provide real areas for reflection regarding the adoption of specific therapeutic strategies with domestic violence abusers.

**Disclosure:**

No significant relationships.

